# Gut Microbiota Improves Prognostic Prediction in Critically Ill COVID-19 Patients Alongside Immunological and Hematological Indicators

**DOI:** 10.34133/research.0389

**Published:** 2024-05-22

**Authors:** Jiaxin Zhong, Li Guo, Yeming Wang, Xuan Jiang, Chun Wang, Yan Xiao, Ying Wang, Fei Zhou, Chao Wu, Lan Chen, Xinming Wang, Jianwei Wang, Bin Cao, Mingkun Li, LiLi Ren

**Affiliations:** ^1^ Beijing Institute of Genomics, Chinese Academy of Sciences, and China National Center for Bioinformation, Beijing, China.; ^2^ University of Chinese Academy of Sciences, Beijing, China.; ^3^ National Health Commission Key Laboratory of Systems Biology of Pathogens, State Key Laboratory of Respiratory Health and Multimorbidity and Christophe Mérieux Laboratory, National Institute of Pathogen Biology, Chinese Academy of Medical Sciences and Peking Union Medical College, Beijing, China.; ^4^ Key Laboratory of Respiratory Disease Pathogenomics, Chinese Academy of Medical Sciences and Peking Union Medical College, Beijing, China.; ^5^ Department of Pulmonary and Critical Care Medicine, Center of Respiratory Medicine, China-Japan Friendship Hospital, Capital Medical University, Beijing, China.; ^6^ National Center for Respiratory Medicine, State Key Laboratory of Respiratory Health and Multimorbidity, National Clinical Research Center for Respiratory Diseases, Institute of Respiratory Medicine, Chinese Academy of Medical Sciences, Department of Pulmonary and Critical Care Medicine, Center of Respiratory Medicine, China-Japan Friendship Hospital, Beijing, China.

## Abstract

The gut microbiota undergoes substantial changes in COVID-19 patients; yet, the utility of these alterations as prognostic biomarkers at the time of hospital admission, and its correlation with immunological and hematological parameters, remains unclear. The objective of this study is to investigate the gut microbiota's dynamic change in critically ill patients with COVID-19 and evaluate its predictive capability for clinical outcomes alongside immunological and hematological parameters. In this study, anal swabs were consecutively collected from 192 COVID-19 patients (583 samples) upon hospital admission for metagenome sequencing. Simultaneously, blood samples were obtained to measure the concentrations of 27 cytokines and chemokines, along with hematological and biochemical indicators. Our findings indicate a significant correlation between the composition and dynamics of gut microbiota with disease severity and mortality in COVID-19 patients. Recovered patients exhibited a higher abundance of *Veillonella* and denser interactions among gut commensal bacteria compared to deceased patients. Furthermore, the abundance of gut commensal bacteria exhibited a negative correlation with the concentration of proinflammatory cytokines and organ damage markers. The gut microbiota upon admission showed moderate prognostic prediction ability with an AUC of 0.78, which was less effective compared to predictions based on immunological and hematological parameters (AUC 0.80 and 0.88, respectively). Noteworthy, the integration of these three datasets yielded a higher predictive accuracy (AUC 0.93). Our findings suggest the gut microbiota as an informative biomarker for COVID-19 prognosis, augmenting existing immune and hematological indicators.

## Introduction

Coronavirus disease 2019 (COVID-19) remains an ongoing challenge for public health 4 years after its initial emergence. COVID-19 frequently manifests respiratory symptoms including cough and difficulty breathing, alongside gastrointestinal symptoms like nausea, vomiting, and diarrhea. Previous studies suggest that 15% to 20% of patients experience gastrointestinal symptoms after contracting the virus [1], with affected patients more prone to severe conditions and extended recovery duration [2].

The gut microbiota is pivotal in modulating the host’s innate and adaptive immune responses to infection, facilitating the proliferation of immune cells and preserving the gut barrier’s integrity. Notably, changes in gut microbiota have been documented in various infectious diseases, including *Salmonella* [3], HIV [4], and influenza [5]. Several studies have indicated a potential link between gut dysbiosis and the severity of bacterial or viral infections in animal models [5,6]. The gut microbiota may affect the respiratory system by producing cytokines, metabolites, and endotoxins that can enter the bloodstream, contributing to the gut–lung axis [7,8]. A recent study has demonstrated changes in the gut microbiota of COVID-19 patients, characterized by a rise in opportunistic pathogens and a reduction in commensal bacteria that produce short-chain fatty acids (SCFAs) [9]. These alterations remain even after the clearance of SARS-CoV-2 RNA and are associated with post-acute COVID-19 syndrome [10]. Additionally, recent studies have shown a relationship between gut microorganisms and the levels of inflammatory cytokines. There is a notable decrease in anti-inflammatory microorganisms, such as *Faecalibacterium prausnitzii* and *Eubacterium rectale*, in COVID-19 patients, which corresponds with elevated levels of tumor necrosis factor (TNF), interleukin-10 (IL-10), and CXCL10 [11]. However, the causality between gut microbiota and COVID-19 was still not entirely clear.

Although the latest SARS-CoV-2 Omicron variant has been found to have decreased pathogenicity compared to previous variants, COVID-19 can still be lethal for the elderly and those with underlying health conditions [12,13]. Thus, accurate identification of high-risk patients using prognostic markers is crucial for intensive treatment and monitoring, potentially reducing mortality. While previous studies have explored the potential of gut microbiota as a prognostic indicator [14], there is still a lack of critical information. First, most studies focused on predicting disease severity rather than mortality. We only found one study assessing the potential of gut microbiota in predicting mortality in COVID-19 patients (with 17 patients who died and less than 100 patients who recovered) [15]. Second, the samples were not collected at uniform time points. This lack of standardized sampling protocols may introduce confounding factors, such as medical intervention, that can affect the accuracy of the results. Third, the changes in marker levels during the course of the disease remain unclear, impeding the inference of causality. Fourth, the relationship between gut microbiota and other biomarkers (e.g., hematological parameters and cytokines) remains largely unknown, making it difficult to effectively integrate possible gut microbiota markers with other markers.

To address the aforementioned concerns, our study conducted a longitudinal cohort study focusing on the gut microbiota of critically ill COVID-19 patients. This study encompassed a cohort of 192 patients in critical condition due to COVID-19, with 39 of these patients unfortunately succumbing to the disease. Anal swabs (ASs) and blood were collected consecutively starting from the first day after admission and subjected to metagenomic sequencing, cytokine measurement, and hematological tests. Our investigation offers a thorough insight into the interplay between the gut microbiota, immune response, hematological parameters, and mortality in critically ill COVID-19 patients. Furthermore, the application of a machine learning model to integrate multidimensional data facilitated an accurate prediction of mortality among COVID-19 patients, achieving an area under the receiver operating characteristic (ROC) curve (AUC) of 0.93.

## Results

### Study design and sample overview

**Fig. 1.**
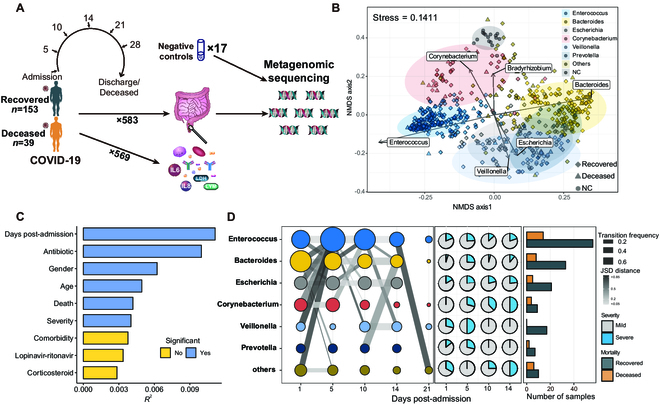
Project design and an overview of the gut microbiota. (A) Schematic diagram of study design. (B) NMDS plot of gut microbiota in patients and NC samples based on the Jensen–Shannon divergence (JSD) distance at the genus level. The samples were colored by CSTs or NC, with arrows highlighting seven genera that explain the highest variance in envfit analysis. The colored shaded areas represent the 95% confidence interval for the CSTs and NC. (C) Variables that correlated with the gut microbiota. The *x* axis shows the *R*^2^ calculated by PERMANOVA using JSD distance, and the color of the bar indicates statistical significance. (D) Transitions between different CSTs during hospitalization. The connecting lines indicate transitions between two CSTs at two consecutive time points. The thickness of the line represents the probability of transfer, while the color of the lines indicates the median JSD distance between the two CSTs. The pie chart displays the proportion of severe COVID-19 patients for each CST at each time point, while the bar chart on the right represents the distribution of mortality for each CST based on the last sample collected for each patient.

Metagenome sequencing was performed on 583 AS samples collected from 192 critically ill COVID-19 patients on days 1, 5, 10, 14, 21, and 28 after admission (Fig. 1A). All participants tested positive for SARS-CoV-2 via reverse transcription polymerase chain reaction (RT-PCR), and they all exhibited pneumonia as confirmed by chest imaging, and had an oxygen saturation (Sao_2_) of 94% or lower on room air, or a Pao_2_:Fio_2_ ratio at or below 300 mmHg. The overall mortality rate in the cohort was 20%, with 39 of 192 patients deceased. Additional demographic and clinical characteristics of the cohort can be found in a previous study on this cohort [16]. Deionized water served as a negative control (NC; *n* = 17) and was handled using the same protocol as the clinical specimens. Blood samples were collected simultaneously with all AS samples, and the concentration of 27 cytokines and hematological parameters were successfully obtained for 582 and 569 blood samples, respectively.

In the cohort, 75.5% (145 of 192) of patients were administered cefoperazone and tazobactam sodium, while 49.5% (96 of 192) received moxifloxacin. Other antibiotics were only administered to 7 to 18 patients. Additionally, approximately 30.2% (58 of 192) of the patients were treated with corticosteroids, including methylprednisolone and prednisone, among others. The sampling time points and the timing of medication administration for each patient are illustrated in Fig. S1. In all subsequent analyses, we included these factors as confounders to mitigate the effect of medication on gut microbiome composition.

### Characterization of the gut microbiota in critically ill COVID-19 patients

In the gut microbiota of COVID-19 patients, bacteria were the predominant microorganisms, representing 96.6% of the microbial reads, followed by fungi (1.9%), archaea (1%), and viruses (0.5%). The microbial composition in these patients can be categorized into six community state types (CSTs) based on the dominant bacteria genus, including communities enriched with *Enterococcus* (37.6% of samples belong to this CST), *Bacteroides* (24.7%), *Escherichia* (12.2%), *Corynebacterium* (8.6%), *Veillonella* (5.3%), and *Prevotella* (4.6%), which formed distinct clusters in the nonmetric multidimensional scaling (NMDS) plot (Fig. 1B and Fig. S2A). Alpha diversity among different CSTs was different, with *Bacteroides*, *Veillonella*, and *Prevotella* CSTs showing significantly higher Shannon indices than *Enterococcus*, *Escherichia*, and *Corynebacterium* CSTs (Fig. S2B).

The gut microbiota composition in COVID-19 patients showed significant differences compared to that of healthy controls, which were collected from the same city and had similar ages in a previous study [*R* = 0.2, *P* < 0.001, analysis of similarities (ANOSIM) test]. The microbiota in 37.6% of COVID-19 samples belonged to *Enterococcus* CST, whereas the microbiota in healthy controls were mainly dominated by *Bacteroides* (89.7%; Fig. S2A). The gut microbiota in COVID-19 patients exhibited a significantly lower alpha diversity (Fig. S2C), a higher abundance of *Enterococcus* and *Methanobrevibacter*, and a lower abundance of *Bacteroides*, *Faecalibacterium*, *Megamonas*, and *Alistipes* compared to healthy individuals (Fig. S2D)*.*

The gut microbiota significantly changed after admission, with the most notable changes occurring between the first and second time points (Fig. 1D and Fig. S2E). Half (44 of 88) of the CST transitions (excluding self-transition) from day 1 to day 5 involved a shift from non-*Enterococcus* CSTs to *Enterococcus* CST, resulting in an increased proportion of *Enterococcus* CST on day 5 (25.3% to 46.5%; *P* < 0.05, Fisher’s exact test). Notably, *Enterococcus*-dominant enterotype was rarely observed in the Chinese population [17]; the shift in CST might be linked to the use of antibiotics, as a significant correlation was found between the abundance of *Enterococcus* and the duration of antibiotic use (Rho = 0.41, *P* < 0.001, Spearman correlation); and *Enterococcus* is known for its resistance to various antibiotics [18].

### The relationship between microbiota composition and mortality

Apart from the well-known correlations of age, gender, and antibiotic treatment with the gut microbiota, our findings revealed a significant correlation between the microbiota composition and both mortality and disease severity among COVID-19 patients (Fig. 1C). The *Bacteroides*, *Veillonella*, and *Prevotella* CSTs were associated with a lower proportion of severe cases (severity score > 4) compared to other CSTs (9.7% to 11.1% versus 20.1% to 29.3%, *P* < 0.05; Fig. 1D). The microbiota of nontypical CST (Others CST), which was predominated by potential pathogens, such as *Candida*, *Klebsiella*, *Staphylococcus*, and *Mycoplasma* (Fig. S2F), exhibited the highest rates of severe conditions and mortality (severe cases: 29.3%, mortality: 26.8%, *P* = 0.056, *P* < 0.05 compared to other samples, Fisher’s exact test). Notably, the *Veillonella* CST was associated with a remarkably low mortality rate (*P* < 0.05, Fisher’s exact test).

**Fig. 2.**
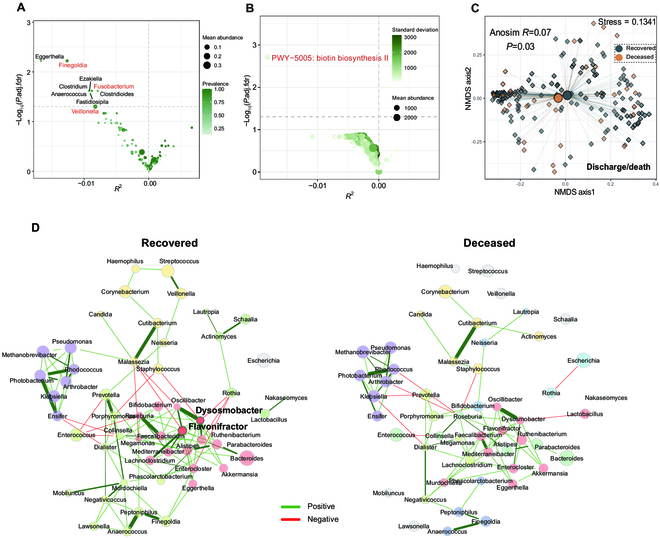
Gut microbiota differences between recovered and deceased patients. (A) Microorganisms with differential abundances in recovered and deceased patients upon admission. The differential abundance analysis was conducted using ZicoSeq when controlling covariates including antibiotics usage, age, gender, disease severity, and corticosteroid usage. The genus abundance matrices were used as the input file. The *x* axis represents *R*^2^, an effect size measure indicating the strength of association with mortality. Genera with significantly different abundances (between recovered and deceased patients) are highlighted in red (Wilcoxon signed-rank test). Genera shown on the left were enriched in recovered patients. (B) Functional pathways with differential abundance in recovered and deceased patients upon admission. The pathway abundance [reads per kilobase (RPK)] matrices were used as the input file. Pathways shown on the left were enriched in recovered patients. (C) NMDS plot of gut microbiota in samples before discharge/death. Recovered and deceased patients were labeled in different colors, with larger dots indicating the center of gravity. The statistical significance of the difference between the microbiota of recovered and deceased patients was calculated using ANOSIM. (D) Comparison of the bacterial interaction in the gut microbiota between recovered and deceased patients at the time of admission. The microbial association network was calculated using the SPRING method. The node size denotes the mclr-transformed abundance. Two hub bacteria (with eigenvector centrality above the 95% quantile) identified in the recovered network were labeled in bold. Node colors represent clusters determined using greedy modular optimization. Clusters in the recovered and deceased patients that shared at least two taxa are colored the same in two networks. Green edges signify positive associations, and red edges indicate negative associations. The same layout is applied to both networks, and nodes not connected in both networks were removed.

Furthermore, by conducting differential analyses (ZicoSeq), we discovered that nine genera were enriched in the recovered patients after adjusting for confounders including antibiotics usage, age, gender, disease severity, and corticosteroid treatment (Fig. 2A). Of these, *Veillonella, Fusobacterium,* and *Finegoldia* showed statistically significant differences in abundance, as determined by the Wilcoxon signed-rank test (adjusted *P* < 0.05). Meanwhile, in terms of microbial functional pathways, PWY-5005: biotin biosynthesis II (vitamin B7), predominantly contributed by *Veillonella*, was significantly increased in the recovered patients (adjusted *P* < 0.05; Fig. 2B). In contrast, we did not observe the enrichment of specific microorganisms or functional pathways in deceased patients, suggesting a lack of consistent disruption.

Then, we focused on two critical cross-sectional time points: the time of admission and discharge/death. These corresponded to the first sample collected within the first 2 days of admission and the last sample collected within 1 week before the patient was discharged or deceased. Although no significant difference in alpha diversity was observed between recovered and deceased patients at the two time points, a notable difference was found in microbiota composition at discharge/death (*R* = 0.07, *P* = 0.03, ANOSIM test; Fig. 2C) but not at admission (*R* = 0.07, *P* = 0.08; Fig. S3A). The abundance of *Veillonella* and biotin biosynthesis pathway was higher in recovered patients compared to deceased patients before discharge/death after adjusting for covariates, whereas no differential microorganisms or pathways were observed upon admission (adjusted *P* < 0.1; Fig. S2B and C).

Microbial network analysis revealed that the community network in recovered patients upon admission exhibited greater edge density (0.084 versus 0.052), higher natural connectivity (0.026 versus 0.023), and hub nodes (eigenvector centrality values above the empirical 95% quantile of all eigenvector centralities) (2 versus 0), suggesting a higher degree of complexity and interactivity within the microecology of those who recovered (Fig. 2D). Furthermore, recovered patients possess a commensal bacterial module (colored red in Fig. 2D), with 11 of the 16 genera in the module being core gut microbiota [19] (Table S1). In deceased patients, these bacteria exhibited lower connectivity and did not form a co-abundant module (connections 6 versus 4, *P* = 0.03). Notably, a similar trend was also observed before discharge/death (Fig. S3D), indicating a more severe disruption of the gut microecosystem in deceased patients.

### The association of gut microbiota with cytokines and hematological parameters in COVID-19

To investigate microbe–host interactions, we analyzed the correlation between gut microbiota and the levels of 27 cytokines (*n* = 582) and 17 hematological parameters (*n* = 569) that were measured at different time points. We observed insignificant correlations between the gut microbiota and cytokine levels, as well as with hematological parameters (*P* > 0.05, Mantel test; Fig. S4A).

**Fig. 3.**
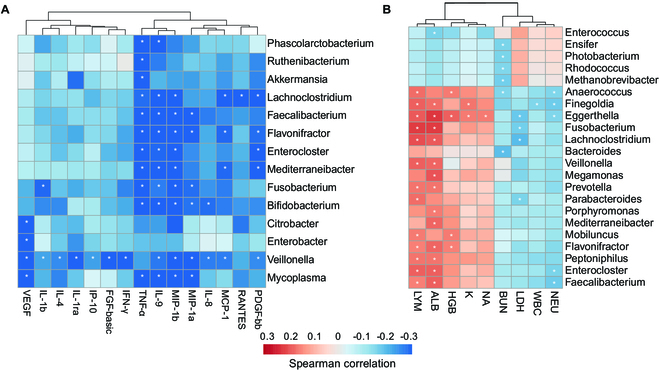
Correlation between gut microorganisms and host cytokines as well as hematological parameters. (A) Partial Spearman correlations between the abundance of microorganisms and the level of cytokines in antibiotic-free samples. (B) Partial Spearman correlations between the abundance of microorganisms and hematological parameters in all samples. White asterisks highlight significant correlation with *P* < 0.05 after using the Benjamini–Hochberg false discovery rate (FDR) correction method. The color reflects the correlation strength and direction in terms of the partial Spearman correlation, adjusting for covariates.

Considering the potential influence of antibiotic administration on the gut microbiota, we restricted the analysis to antibiotic-naïve samples (*n* = 91) obtained from relatively mild patients (with severity scores between 3 and 5). This subset demonstrated a notable correlation between gut microbiota and cytokine level (*r* = 0.12, *P* < 0.001; Fig. S4A). Particularly, *Veillonella* showed a negative correlation with several proinflammatory cytokines [IL-1ra, IL-1b, IL-8, interferon-γ (IFN-γ), and IP-10; Fig. 3A]. Meanwhile, significant negative correlations were identified between the presence of commensal bacteria like *Bifidobacterium*, *Faecalibacterium*, and *Akkermansia*, and various cytokines (TNF-α, IL-9, MIP-1α, MIP-1β), pointing toward a healthier gut microbiota being linked to reduced inflammatory status. Remarkably, in mild cases, no positive correlations were found, whereas a positive correlation was observed between the abundance of *Candida* and a proinflammatory cytokine IL-17 in the complete datasets, which encompassed severely ill patients (Fig. S3B).

Concerning the correlations between hematological parameters and individual microorganisms, we found that the levels of LYM (lymphocyte ratio) and ALB (albumin blood) exhibited positive correlations with several gut commensal bacteria in the complete datasets (Fig. 3B), including *Faecalibacterium*, *Prevotella*, and *Fusobacterium*. Conversely, BUN (blood urea nitrogen), LDH (lactate dehydrogenase), and NEU (neutrophil ratio) showed negative correlation with specific gut commensal bacteria, including *Bacteroides*, *Faecalibacterium*, and *Fusobacterium*. Meanwhile, we also found that the levels of two organ damage markers LDH and ALB were correlated with the abundance of numerous microbial functional pathways, including those related to amino acid metabolism and tricarboxylic acid (TCA) cycle (Fig. S4C). Of note, the biotin metabolism pathway, previously identified to be enriched in recovered patients, exhibited a positive correlation with the level of ALB.

### Inflammatory cytokines and hematological parameters were correlated with COVID-19 severity and mortality

**Fig. 4.**
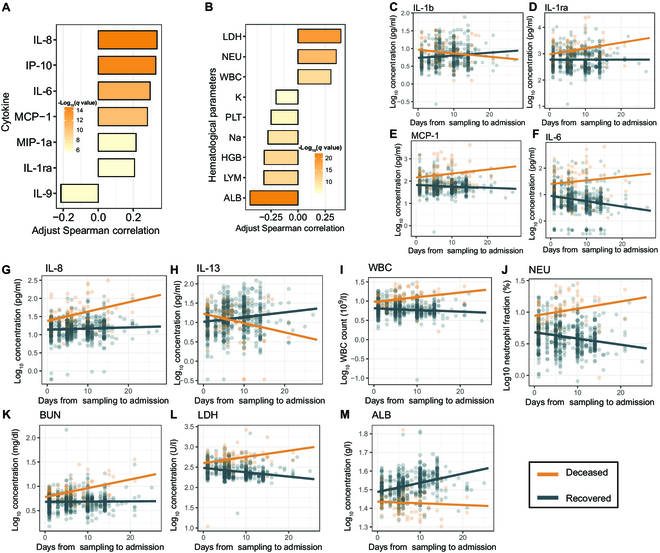
Immunological and hematological parameters correlated with clinical outcomes in COVID-19 patients. (A) Cytokines significantly correlated with disease severity. Cytokines with an absolute partial Spearman correlation (after adjusting for covariates) greater than 0.2 are shown (adjusted *P* < 0.05). (B) Hematological parameters significantly correlated with disease severity. Hematological parameters with an absolute partial Spearman correlation greater than 0.2 are shown (adjusted *P* < 0.05). (C to M) Dynamics of cytokines and hematological parameters display statistically significant differences in trends among patients with different clinical outcomes during hospitalization. The line indicates the regression line.

Seven cytokines showed a significant positive correlation with disease severity (|Rho| > 0.2, partial Spearman correlation), including proinflammatory T helper 1 (T_H_1)-related cytokines (IP-10, MIP-1a) and acute respiratory distress syndrome (ARDS)-associated cytokines [20] (IL-6, IL-8, IL-1ra, MCP-1) (Fig. 4A). Conversely, only IL-9 showed a negative correlation with disease severity. Meanwhile, among the 17 hematological parameters examined, LDH, NEU, and WBC (white blood count) were positively correlated with the disease severity, while K, Na, HGB (hemoglobin count), PLT (platelet count), LYM, and ALB were negatively correlated with the disease severity (Fig. 4B).

We then examined the correlation between the dynamics of cytokines and hematological parameters during the hospitalization and the clinical outcomes. Six cytokines (IL-1b, IL-1ra, IL-6, MCP-1, IL-8, IL-13) and five hematological parameters (WBC, NEU, BUN, LDH, ALB) showed distinct trends in the recovered and deceased patients (*P* < 0.05, linear mixed-effects model, differential trend analysis; Fig. 4C to M). We noted that most of the markers were also correlated with the disease severity in the previous cross-sectional analysis (Fig. 4A and B). All of these markers showed a significant increase in the deceased patients, while they either decreased or remained stable in the recovered patients, except for three markers (IL-1b, IL-13, and ALB), which showed a reverse trend. In contrast, we did not find any microorganisms that showed a significant correlation with the clinical outcome in the differential trend analysis after correction for multiple comparisons.

### Improved predictive power through the integration of metagenomic, hematological, and cytokine data

**Fig. 5.**
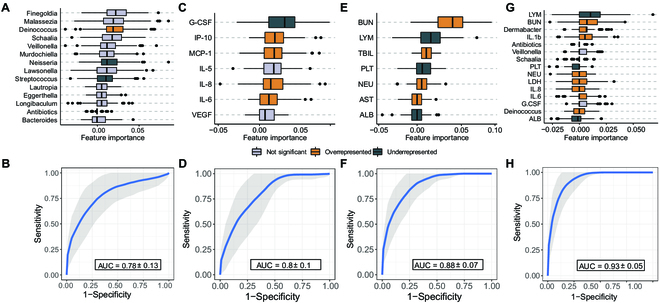
Performance and discriminative features of random forest classifiers for predicting clinical outcome. Discriminative features selected by the models and ROC curves for each random forest classifier to predict clinical outcomes. Gut microbiota (A and B), cytokines (C and D), hematological parameters (E and F), and a combination of all three datasets (G and H) are displayed. The colors of the boxplot indicate the statistical significance of the abundance differences between deceased and recovered patients (*P* < 0.05, Wilcoxon signed-rank test). The AUC value is derived from 1000 randomized trials using fivefold cross-validation.

The observed correlation between the gut microorganisms, cytokines, and hematological parameters and the clinical outcome implies their potential as prognostic biomarkers in COVID-19 patients. We developed imbalanced random forest classifiers for predicting clinical outcomes (recovery or deceased) based on the data collected in the first 2 days after admission (*n* = 124). Seven models were constructed based on the gut microbiota composition, cytokine concentration, and hematological parameters, both independently and in combination with the other datasets. The microbiota composition-based classifier exhibited the poorest performance among all models, with an AUC of 0.78 (Fig. 5A), yet surpassed prediction based on the clinical severity score (the seven-category ordinal scale) (AUC 0.64). This model identified 12 genera as distinct features (Fig. 5B), with three of them (*Deinococcus*, *Neisseria*, and *Streptococcus*) showing significant differences between recovered and deceased patients (*P* < 0.05, Wilcoxon signed-rank test). In addition, the prediction model based on the abundance of microbiota functional genes demonstrated inferior performance compared to the microbiota composition-based model (AUC = 0.72). In contrast, cytokines and hematological parameters provided a better performance (AUC = 0.80 and 0.88, respectively), with seven cytokines and seven hematological parameters selected as distinct features in the model (Fig. 5C to F). Among these distinct features, five of seven of the cytokines and seven of seven of the hematological parameters were significantly different between recovered and deceased patients.

Multidimensional models outperformed those based on single datasets. Specifically, the combination of hematological and cytokines data produced an AUC of 0.89 (Fig. S5A and B), while cytokines and microbiota data resulted in an AUC of 0.85 (Fig. S5C and D), and hematological and microbiota data yielded an AUC of 0.9 (Fig. S5E and F). Furthermore, the model incorporating all three datasets achieved the highest AUC of 0.93 (Fig. 4G and H). Other performance metrics including F1 scores, precision, recall, and accuracy were summarized in Table S2. It is worth noting that the distinct features identified in the best-performing model, which incorporated all three datasets, included a combination of immunity markers [e.g., LYM, NEU, IL-8, IL-6, granulocyte colony-stimulating factor (G-CSF), and IL-1b], organ damage markers (e.g., BUN, LDH, and ALB), and microbial markers (*Dermabacter*, *Deinococcus*, *Veillonella*, and *Schaalia*). These results suggest that a diverse set of markers are complementary in predicting the clinical outcome, and their combination can lead to a more accurate and comprehensive prediction model.

Besides the time point of admission, we also developed predictive models for samples taken on the fifth day after admission and at discharge/death (models for other time points were not constructed due to the small number of deceased patients). We observed a decline in the predictive capability of the microbiota on the fifth day after admission, with an AUC of 0.71. Conversely, cytokines and blood markers showed higher predictive power, with AUCs of 0.92 and 0.95, respectively, and the multidimensional data model reached an AUC of 0.96 (Fig. S6). Furthermore, at the time of discharge/death, all models showed improved predictive performance. The microbiota model had an AUC of 0.81, while cytokines and blood markers achieved AUCs of 0.96 and 0.98 (Fig. S7), respectively. However, these samples were collected within 1 week before discharge/death, which might limit their values in a clinical practice.

## Discussion

Since the beginning of the COVID-19 pandemic, numerous studies have reported that SARS-CoV-2 infection disrupts host immune and intestinal homeostasis, with aggressive inflammatory responses identified as a prominent factor contributing to severe disease outcomes and mortality [21]. Gut commensal flora is acknowledged for enhancing host immunity against pathogens and regulating inflammatory responses, thereby aiding in infection prevention and disease control [22,23]. In our study, we identified a significant difference in microbial composition and diversity between COVID-19 patients and HCs, which indicated a dysbiosis of the gut microbiota in COVID-19 patients. Previous COVID-19 studies have demonstrated the depletion of *Faecalibacterium* and *Bacteroides* [24], and the enrichment of *Enterococcus* [25], which was consistent with the findings in our study (Fig. S2D).

While a previous study involving 95 COVID-19 patients (including 42 deceased patients) reported that the increased abundance of *Enterococcus* in rectal swabs was associated with a higher mortality risk [26], our study did not observe a similar correlation. We speculate that the *Enterococcus* abundance increase might be linked to antibiotic usage, especially within the first 5 days of administration. This is consistent with prior findings that have highlighted *Enterococcus*’s resistance to various antibiotics, including β-lactam and vancomycin, which were frequently prescribed in our cohort [18]. Moreover, while several studies have noted an increase in the abundance of *Veillonella* in COVID-19 patients [27–29], with one even suggesting a link between *Veillonella* and more severe COVID-19 symptoms, our study presented different findings. Specifically, we discovered that the presence of *Veillonella* was associated with improved clinical prognosis. Recent literature suggests that *Veillonella* can modulate the immune system’s response to viral infections by producing beneficial metabolites, SCFAs, through its lactate metabolism function, which results in the generation of propionate and acetate [30–32]. Interestingly, we found a significant positive correlation between the abundance of *Veillonella* and levels of propionate (rho = 0.26, adjusted *P* < 0.001) and butyrate (rho = 0.32, adjusted *P* < 0.001) inferred from the metagenomic data using MelonnPan [33]. These SCFAs could strengthen gut integrity and immune function, thereby potentially reducing the severity of COVID-19. *Veillonella* also plays a significant role in biotin synthesis, which might suppress the expression of proinflammatory cytokines, and potentially plays a key role in the COVID-19 recovery process [34,35]. Notably, our study found a relatively weak association between gut microbiota and disease severity compared to previous studies. This may be attributed to a more significant perturbation in the gut microecosystem of critically ill patients, possibly resulting from disease progression, medical intervention, or a combination of both factors.

Proinflammatory T_H_1-related cytokines (IP-10 and MIP-1a) and ARDS-associated cytokines (IL-6, IL-8, IL-1ra, and MCP-1) [20] showed positive correlations with the severity of COVID-19. In addition, hematological parameters, including blood organ damage indicators (LDH and ALB) and immune cell indicators (NEU, LYM, and WBC) also strongly correlated with disease severity, consistent with prior studies [2,21,36]. Moreover, we found that nearly half of these factors exhibited opposite dynamic trends in different clinical outcomes of COVID-19 over time, further validating the efficacy of these biomarkers for severe cases and prognosis prediction. Meanwhile, significant correlations were observed between gut microorganisms and cytokines, suggesting an intense interaction between the host and the gut microbiota. Specifically, *Veillonella* showed a significant negative correlation with over 10 inflammation-related cytokines, underscoring its potential role in mitigating inflammation. Additionally, significant negative correlations were found between various gut commensals, including *Akkermansia*, *Flavonifractor*, *Lachnoclostridium*, *Faecalibacterium*, and *Bifidobacterium*, and inflammatory cytokines such as TNF-α, MIP-1α, and IL-8. Notably, these commensal bacteria formed a closely interconnected network in the gut microbiota of recovered patients but not in those who were deceased. This indicates a close relationship between the balance of gut microbiota and the body’s inflammation levels. While the exact causality is unclear and likely complex, dysbiosis in the gut microbiota has the potential to compromise lung immunity or even impair antiviral immunity through alterations in gut microbiota components (e.g., peptidoglycan) or metabolites (e.g., SCFA) that are translocated to the lung via the circulatory system [8]. Understanding these interactions can offer novel insights into strategies for modulating immune responses in COVID-19 patients.

Predicting the risk of patients as early as possible is essential for improving clinical outcomes. Gut microbiota has been proposed as a potential noninvasive prognosis marker [15]. However, the performance is inflated as the previous study used samples that had already developed severity, rather than the sample collected on admission. In this study, we achieved an AUC of 0.76 to predict mortality using samples collected on admission. The AUC could be further increased to 0.79 when simultaneously considering the severity score on admission, in contrast to 0.64 when only using the severity score, underscoring the substantive value of gut microbiota in practical prognostication of COVID-19. More importantly, our study as the inaugural study to leverage multidimensional data for predicting clinical outcomes in patients with infectious diseases highlighted a complementary value of gut microbiota in conjunction with hematological parameters and cytokines, which are widely accepted as useful prognostic biomarkers, for predicting the risk of the disease (AUC = 0.92). The superior performance of the prediction model, which combined blood, cytokines, and gut microbiota data, underscores the benefits of multimodal data integration, a concept gaining prominence in disease diagnosis and other applications.

There are some limitations to our study. First, the profound impact of the antibiotics administered in this critically ill cohort on the microbiota might have obscured certain correlations. To mitigate the influence of antibiotics, we have incorporated the use of antibiotics into the analysis. Additionally, our study centered on the analysis of samples collected within the initial 2 days after admission, a period during which the impact of antibiotics is minimal. Second, our samples were collected during the pandemic’s initial stages, from patients infected with the original SARS-CoV-2 strain, whose pathogenic properties differ from those of the later-emerging Omicron variants. Thus, the characteristics and predictiveness of the gut microbiota may vary between different variants. Meanwhile, as the composition of the gut microbiome also significantly varies across different populations due to dietary habits, genetics, and environmental factors [37], the performance of our predictive model on other populations is unclear, especially considering that the microorganisms associated with the severity of COVID-19 varied in different studies that enrolled different populations [38]. Future research that includes diverse populations is warranted to ascertain the generalizability of the findings and models in this study.

### Conclusions

Our study employed a metagenomic approach to longitudinally characterize the profile of the gut microbiota in critically ill COVID-19 patients, along with host cytokines and hematological parameters. We observed a correlation between dysbiosis of the gut microbiota and disease severity as well as mortality. The gut microbiota upon admission exhibited a moderate predictive capacity for clinical outcomes. Moreover, integrating cytokine, hematological parameters, and gut microbiota data significantly improved prediction accuracy compared to models based on individual datasets, emphasizing the crucial role of comprehensive data integration in advancing our understanding and predictive capabilities related to clinical outcomes.

## Materials and Methods

### Subject recruitment and sample collection

COVID-19 patients were enrolled in the Lopinavir Trial for Suppression of SARS-CoV-2 in China (LOTUS) (ChiCTR2000029308) [16]. The mortality refers to the outcome of our continuous tracking of these patients, where the patients either recovered and were discharged or died from respiratory failure, and the median hospital stay for deceased patients was 11 days, with an interquartile range (IQR) of 7 to 15.5 days. To enhance convenience for critically ill patients, we opted for ASs as an alternative to stool samples. The feasibility of exploring the gut microbiota using ASs has been confirmed in previous studies [39]. AS samples were collected on days 1, 5, 10, 14, 21, and 28 after admission. Additionally, a set of health control data was collected from a previous study conducted in the same geographic region (Hubei Province) [40].

### DNA extraction and sequencing

DNA from AS specimens was extracted using the NucliSENS easyMAG (BioMerieux, France). Metagenomic libraries were prepared utilizing the TruePrep DNA Library Prep Kit V2 for Illumina (Vazyme, China) following the manufacturer’s protocol. The purification step employed Agencourt AMPure XP beads (Beckman Coulter, USA), and quantification was carried out using Qubit dsDNA Quantification Assay Kits and the Qubit 4.0 Fluorometer (Thermo Fisher Scientific, USA). Sequencing took place on the Illumina NovaSeq 6000 platform in paired-end mode with 2 × 150–base pair (bp) reads (six-gigabyte data per sample). To mitigate the impact of laboratory-derived contaminants, we incorporated 17 NCs during processing, which included 15 DNA extraction controls and 2 DNA library no-template controls.

### Taxonomic and functional profiling of the metagenomic data

Raw reads were filtered and quality-trimmed using Fastp (version 0.20.1) with the following parameters (-l 50 -x --detect_adapter_for_pe --overlap_len_require 20 --overlap_diff_limit 5 --overlap_diff_percent_limit 20 --cut_tail --cut_tail_mean_quality 15). Contaminating human reads were filtered using bmtagger (version 1.0) with default parameters, and the reference database included the human genome (GRCH38) and UNiVec sequences. As a result, a total of 5 × 10^9^ (8.6 × 10^6^ on average) reads were retained for subsequent analyses.

Bacterial taxonomy profiling was conducted using Megablast (version 2.9.0) [41] (-value 1e-10 -qcov_hsp_perc 60 -perc_identity 60) against the National Center for Biotechnology Information (NCBI) Nucleotide database (version 2020.02.25), followed by MEGAN [42] analysis (-ms 100 –supp 0 –me 0.01 –mrc 60) (version 6.21.16). Only taxa with a relative abundance >0.01 in at least one sample were included in subsequent analyses. Reads assigned to the genus level were reallocated to the species level using Bracken [43]. Humman2 [44] (v0.11.1) was used to quantify microbial functional pathways.

### Plasma cytokine measurements

The human cytokines were screened using the Bio-Plex Pro Human Cytokine Screening Panel, 27-plex (Bio-Rad, USA) on Bio-Plex 200 platform (Bio-Rad, USA). The panel included 27 cytokine and chemokine cell signaling molecules, including FGFbasic, Eotaxin, G-CSF, granulocyte-macrophage colony-stimulating factor (GM-CSF), IFN-γ, IL-1β, IL-1ra, IL-2, IL-4, IL-5, IL-6, IL-7, IL-8, IL-9, IL-10, IL-12 (p70), IL-13, IL-15, IL-17A, IP-10, MCP-1 (MCAF), MIP-1α, MIP-1β, platelet-derived growth factor (PDGF)-BB, RANTES, TNF-α, and vascular endothelial growth factor (VEGF).

### Hematological parameter tests

Hematological parameters, including white blood cell count (WBC), hemoglobin (HGB), platelets (PLT), neutrophil ratio (NEU), lymphocytes (LYM), prothrombin time (PT), activated partial thromboplastin time (APTT), total bilirubin (TBIL), alanine aminotransferase (ALT), aspartate aminotransferase (AST), serum albumin (ALB), blood urea nitrogen (BUN), creatinine (CR), creatine kinase (CK), lactate dehydrogenase (LDH), potassium (K), and sodium (Na), were measured by automated hematology analyzer, clinical biochemistry analyzer, and coagulation analyzer.

### Clustering analysis

Microbiota clustering was performed based on the dominant genus in each sample. Initially, the most abundant genus in each sample was identified, and those observed in more than 10 samples were utilized to define the major CST. If the most abundant genus was not among these dominant genera, the CST was assigned based on the second most abundant genus, provided its abundance surpassed more than half of the most abundant one. Finally, all remaining samples were defined as Others CST. To validate the rationale of our clustering method, we compared it with another two widely used methods, partitioning around medoids (PAM) and Dirichlet multinomial mixture (DMM) clustering methods [45]. The PAM clustering method suggested that our data best fit into two distinct clusters, while the DMM method indicated a division into seven clusters (Fig. S8A and B), with varying dominant species across these groups (Fig. S8C). We calculated the proportion of variance in the gut microbiota explained by different clustering schemes using permutational multivariate analysis of variance (PERMANOVA) analysis. The *R*^2^ value for clustering based on dominant bacteria was 0.71, higher than that of DMM at 0.61 and PAM at 0.36 (Fig. S8D to F). Thus, the clustering method based on dominant bacteria is more suitable for our data due to its stability, reproducibility, and the intuitiveness of sample presentation.

### Machine learning model for clinical outcome prediction

The random forest model was employed for predicting clinical outcomes. To address the uneven sample size in our data, we utilized the synthetic minority oversampling technology (SMOTE) method through the R package imbalance to generate additional data, thereby facilitating a more robust model construction. Feature selection within the random forest model was performed using the R package VSURF [46], which utilizes a permutation-based importance score. Our analysis consisted of 1000 rounds of random resampling and random forest analyses, and average AUC values were utilized in the study.

### Statistical analysis

Nonparametric tests (Mann–Whitney or Kruskal–Wallis test) were used to compare the data across two or more categories. Alpha diversity was computed using the diversity function in the vegan package. We examined differences in slopes of the regression line between various clinical outcomes using the “lstrends” function in R package lsmeans. LEfSe and ZicoSeq [47] analyses (adjusting for covariates including antibiotics usage, age, gender, disease severity, and corticosteroid usage) were performed to identify microorganisms exhibiting differential abundance in two groups. Network analysis was used to explore the microbial community difference between recovered and deceased patients based on R package NetCoMi [48]. Correlations between microbiota, cytokines, and hematological parameters were assessed using partial_Spearman function in R package PResiduals with adjustment for covariates. The Mantel test in package “vegan” was performed to quantify the correlation between each paired distance matrix. The Benjamini–Hochberg method was applied to adjust for the effect of multiple hypothesis testing.

## Ethical Approval

The study was approved by the institutional review board of Jin Yin Tan Hospital (KY2020-02.01). Written informed consent was obtained from all patients or their legal representatives if they were too unwell to provide consent.

## Data Availability

The raw sequence data used in this study were deposited in the GSA database in National Genomics Data Center under the accession number of CRA008777, which is publicly accessible at https://ngdc.cncb.ac.cn/gsa. Analysis code and metadata for each figure are available via GitHub (https://github.com/JX-Zhong/KLZ_AS_analysis).
